# Radiation exposure in recurrent medical imaging: identifying drivers and high-risk populations

**DOI:** 10.3389/fpubh.2025.1626906

**Published:** 2025-07-18

**Authors:** Juan Chen, Jianjun Zheng, Qun Zhang, Jingfeng Zhang, Qi Dai, Dandan Zhang

**Affiliations:** ^1^Ningbo No. 2 Hospital, Ningbo, China; ^2^Hangzhou Medical College, Hangzhou, China; ^3^Ningbo Municipal Center for Disease Control and Prevention, Ningbo, China

**Keywords:** recurrent imaging, radiation exposure, cumulative radiation exposure, dose optimization, radiation protection

## Abstract

Medical imaging modalities constitute indispensable diagnostic and therapeutic decision-making tools in contemporary clinical practice. These modalities are pivotal in disease detection, longitudinal monitoring, and treatment response assessment. However, the progressive accumulation of radiation exposure from recurrent imaging procedures has sparked significant clinical concerns regarding potential carcinogenic and non-carcinogenic health implications. This review analyzes the driving factors of recurrent medical imaging examinations, identifies high-risk populations, and evaluates the potential health risks associated with cumulative radiation exposure, aiming to optimize imaging techniques and dose management strategies. By integrating global radiation exposure data (e.g., UNSCEAR reports) and multicenter clinical research evidence combined with a literature review and dosimetry models, the study reveals the high-risk nature of emergency department patients, chronic disease patients, children, and female populations in recurrent imaging. Clinical needs, demographic characteristics, technological misuse, and uneven healthcare resource allocation are identified as key drivers of recurrent imaging. This review further highlights that short-term, high-frequency imaging accelerates cumulative radiation dose accumulation, potentially elevating long-term health risks, while long-term, low-dose exposure is associated with cardiovascular diseases and malignancies. Based on the linear no-threshold (LNT) model and evidence of DNA repair mechanisms, the study proposes individualized risk assessment to optimize imaging intervals and dose modulation techniques to balance diagnostic efficacy and radiation safety. The policy implications of this research include advocating for enhanced radiation safety education and targeted management strategies for high-risk populations and providing empirical support for updating international radiation protection guidelines, thereby facilitating the clinical implementation of the “As Low As Reasonably Achievable” (ALARA) principle.

## Highlights

- The increasing trend in global radiological examinations has led to a significant increase in cumulative radiation exposure risks, particularly as the proportion of patients undergoing repeated imaging procedures continues to rise.
- Clinical needs (e.g., diagnostic uncertainty), demographic factors (e.g., aging populations), technological advancements (e.g., faster CT scans), and systemic inefficiencies (e.g., suboptimal image quality) are identified as key drivers of repeated imaging.
- Dose modulation techniques, individualized risk stratification, and enhanced radiation safety education are recommended to balance diagnostic efficacy with patient safety, aligning with the ALARA (As Low As Reasonably Achievable) principle and supporting updates to international radiation protection guidelines.

## 1 Introduction

**Figure 1:**
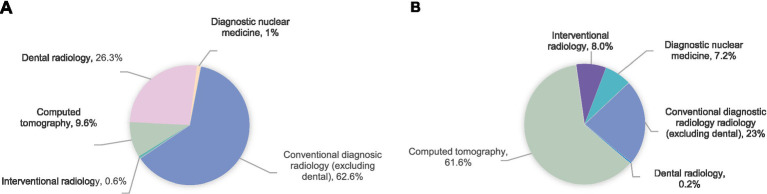
Distribution of examinations/procedures by imaging modality **(A)** and their contribution to the collective effective dose from medical exposures **(B)**.

Medical imaging examinations constitute essential diagnostic and management tools in contemporary healthcare (1). However, the increasing frequency of these procedures has raised global radiation exposure concerns. According to the United Nations Scientific Committee on the Effects of Atomic Radiation (UNSCEAR) 2020/2021 report (2), approximately 4.2 billion medical imaging examinations are conducted worldwide each year, including over 1 billion computed tomography (CT) scans, which account for 10% of the total. Despite comprising only 10% of all procedures, CT contributes to 62.6% of the collective effective dose (3). Figure 1 demonstrates (a) the modality-specific distribution of imaging examinations and (b) their respective contributions to the total effective dose from medical radiation exposure. The increasing trend in global radiological examinations has led to a significant increase in cumulative radiation exposure risks.

Studies indicate that more than 1% of patients have received a cumulative radiation dose exceeding 100 mSv over their lifetime due to repeated imaging examinations (4–7). Prolonged exposure to such high radiation levels can lead to a range of health issues, including cancer, damage to the hematopoietic system, thyroid dysfunction, liver impairment, damage to the ocular lens, and immune system disorders (5–9).

Contemporary evidence demonstrates that recurrent imaging examinations are notably prevalent among specific populations, including emergency department patients, individuals with chronic conditions, and those in intensive care units (10–12). A study involving 2.5 million patients and 4.8 million CT examinations revealed that patients underwent a median of six CT scans per year, with some individuals receiving as many as 109 imaging procedures over 5 years (6). Current research has confirmed that patients undergoing recurrent imaging examinations may put them at an increased risk of cancer due to increased cumulative effective doses (CED) (13). Although organizations such as the International Commission on Radiological Protection (ICRP) have explicitly stated that radiation doses from medical imaging should adhere to the principle of “As Low As Reasonably Achievable” (ALARA) (14), excessive and unnecessary repeated imaging examinations remain prevalent in clinical practice.

The optimization of imaging techniques and dose management strategies for these high-risk populations requires additional investigation. Targeted radiation safety education programs should be implemented for populations requiring recurrent imaging and to formulate tailored imaging strategies through suitable customized approaches and appropriate validation systems (15). This paper aims to systematically analyze the primary driving factors of recurrent imaging examinations, examine the diverse structural characteristics of radiation exposure in repeated imaging procedures, and assess the efficacy of current risk assessment models and methodologies.

While multinational cohorts have quantified recurrent CT risks (5, 6, 16, 17), population-specific evidence from China—where over 1 billion medical imaging examinations are performed annually—remains scarce. Recent data from Shanghai indicate that during the 1.6-year observation period, 78.43% of patients underwent only a single CT examination, but 0.03% underwent more than 10 examinations, of which 0.05% (53 patients) had a cumulative effective dose (CED) of more than 50 mSv, and 1 case had a CED of more than 100 mSv (18) (Table 1).

**Table 1:** Trends in global medical exposures.

Source	Annual no. of examinations (millions)*	Annual frequency of procedures per 1,000 people	Annual collective effective dose (1,000 man Sv)*	Annual per capita dose (mSv)*
UNSCEAR 1988	1,740	355	1,890	0.37
UNSCEAR 1993	1,620	305	1,780	0.33
UNSCEAR 2000	2,460	426	2,460	0.43
UNSCEAR 2008	3,660	561	4,210	0.65
UNSCEAR 2022	4,190	574	4,210	0.58

Source: Thomas (3). *Values were rounded. For the effective dose determination, International Commission on Radiological Protection 60 tissue weighting factors were applied (98).

### 1.1 Definition and classification of recurrent medical imaging

Recurrent medical imaging refers to patients requiring multiple imaging procedures within a short time frame or over different periods to diagnose diseases, assess treatments, or conduct ongoing follow-ups (1, 19). It can be categorized into unplanned (situational) and planned (follow-up) examinations.

Situational examinations are primarily utilized for rapid diagnosis and evaluation of acute conditions. These typically occur in clinical settings such as emergency rooms or trauma departments (20). Situational examinations are necessary when patients present with complex conditions or unclear symptoms, leading to multiple imaging procedures within a short period. For instance, polytrauma patients may undergo combined CT scans of the chest, abdomen, and pelvis to comprehensively assess the extent and severity of their injuries (21). Since the need for quick diagnosis drives these examinations, strict management of radiation doses is essential.

Planned examinations, on the other hand, are used for the regular follow-up of patients with chronic diseases or for monitoring the recurrence of illnesses. These examinations aim to track changes in a patient’s condition over time. For example, cancer patients need regular imaging following chemotherapy or radiotherapy to evaluate treatment effectiveness and detect any recurrence of the disease, while patients with chronic cardiovascular conditions require imaging to monitor stability over time (22). Because these examinations are conducted regularly, radiation exposure can accumulate significantly, especially during prolonged follow-up periods. Therefore, paying careful attention to radiation dose management and optimizing follow-up strategies during these examinations is crucial (19).

## 2 Drivers of recurrent imaging utilization

**Figure 2:**
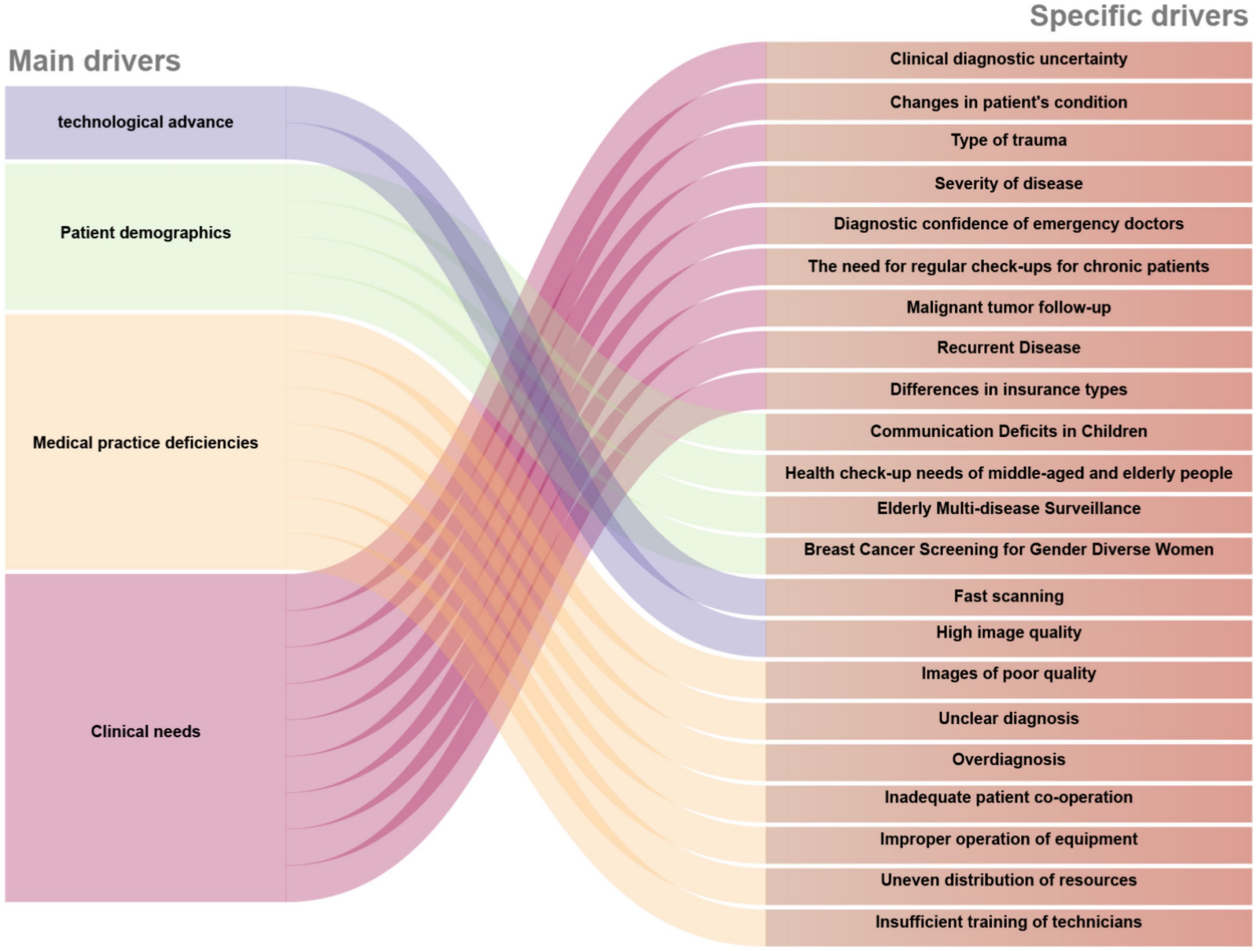
Factors drivers of recurrent imaging utilization.

The increasing use of medical imaging has produced a complex practical environment where clinical needs, demographic factors, and technological advancements intersect, as illustrated in Figure 2. Systemic challenges also influence overall radiation exposure profiles. This section explores four key dimensions through which these driving forces manifest, starting with the essential clinical demands establishing baseline imaging requirements.

### 2.1 Clinical drivers: dual challenges in emergency care and chronic disease management

Patients in emergency departments undergo frequent imaging examinations, particularly those with trauma or acute conditions, as imaging plays a critical role in rapid clinical assessment, diagnosis, and treatment guidance (23, 24). One major factor driving repeated imaging in emergency departments is clinical diagnostic indeterminacy. Clinicians frequently employ repeat imaging to adjudicate equivocal findings from initial examinations, a critical practice in time-sensitive emergency settings where diagnostic certainty directly informs time-critical interventions. Furthermore, the temporal evolution of disease manifestations necessitates serial imaging, as clinicians may mandate repeat examinations to evaluate clinical deterioration or emergent complications following baseline CT assessments. Lee et al. (25) reported a 30% novel pathology detection rate through repeat CT examinations disclosed novel or progressive pathological features, underscoring how clinical trajectory modifications potentiate demands for supplemental imaging. The diagnostic confidence level of emergency physicians may influence decisions to perform repeat imaging. Diagnostic uncertainty arising from equivocal primary imaging findings frequently precipitates repeat examinations to achieve diagnostic resolution. Studies indicate that imaging utilization patterns demonstrate a significant correlation between trauma classification and disease severity (26), particularly among patients with higher Injury Severity Scores (ISS), who exhibit an increased likelihood of repeat imaging. Significant heterogeneity in repeat CT utilization has been documented across insurance status cohorts, with commercially insured patients demonstrating distinct patterns compared to uninsured counterparts (23).

Chronic disease populations and individuals with recurrent pathologies demand recurrent imaging surveillance to address longitudinal management requirements and therapeutic monitoring (27, 28). As exemplified in oncological care, imaging modalities constitute an indispensable component of post-therapeutic surveillance, treatment efficacy adjudication, and malignancy recurrence detection protocols (29). Epidemiologic data indicate that 90% of individuals with a cumulative effective dose (CED) of 100 mSv or more are diagnosed with malignancies. This clearly identifies chronic and recurrent disease cohorts as radiation-sensitive subpopulations for cumulative radiation exposure. Additionally, non-oncological chronic conditions, such as cardiopulmonary diseases and chronic obstructive pulmonary disorders, represent a significant portion of recurrent imaging utilization patterns (30).

### 2.2 Demographic characteristics of patients: significant impact of age and gender

The frequency of imaging examinations varies significantly among patients of different age groups (31). Children frequently undergo CT or X-ray examinations to monitor growth and development and manage acute trauma. Developmental stage-specific communication barriers may result in unnecessary CT scans (32). The older adult population requires more imaging examinations due to the increased prevalence of multiple chronic diseases, necessitating disease progression and treatment efficacy assessments. Middle-aged and older adult patients exhibit a greater demand for imaging examinations in health monitoring (33).

Female patients, particularly those undergoing breast cancer screening and follow-up, require regular imaging examinations. According to the guidelines of the American Cancer Society, women over the age of 40 should undergo annual mammography, while high-risk groups, such as those with BRCA1 or BRCA2 gene mutations, require more frequent screenings (1). Furthermore, breast cancer recurrence monitoring requires patients to undergo imaging follow-ups every 3–6 months after surgeries (34). Moreover, psychological factors such as health anxiety may prompt some women to request repeat examinations more frequently. These women often exhibit heightened concern about disease risk, leading them to request additional checks even in low-risk scenarios.

### 2.3 Technological advancements: enhanced efficiency and dose traps

Recent advancements in imaging technologies have led to significant reductions in scan time and substantial improvements in image quality, thereby driving the widespread adoption of imaging examinations (35, 36). The proliferation of helical CT technology has resulted in faster scan speeds and extended coverage, consequently driving a significant rise in the frequency of CT examinations. However, these technological advancements have given rise to misconceptions, particularly the belief that reduced scan time correlates with lower radiation doses. In fact, the increased scan coverage has led to the oversight of cumulative radiation exposure. Therefore, although technological progress has enhanced diagnostic sensitivity, increasing radiation doses remains a concern (35). Particularly in pediatric cases, fast scanning and superior image quality reduce the need for sedative, thereby enhancing the acceptance of imaging procedures (37).

### 2.4 Deficiencies in medical practice: from quality control to resource allocation

Certain inappropriate practices in clinical settings may lead to unnecessary repeat imaging examinations. Specifically, suboptimal image acquisition parameters and indeterminate diagnostic interpretations, or overdiagnosis may result in patients undergoing repeated imaging examinations, thus increasing the risk of radiation exposure (30). Quality control deficiencies also contribute significantly, as disparities in imaging quality—such as blurred images or misdiagnosis—may force patients to undergo repeat scans to confirm diagnostic accuracy (38). Poor patient cooperation or improper equipment operation during imaging procedures may also result in repeat examinations. For instance, patients with involuntary motion artifacts or improper use of equipment may necessitate repeat imaging (35, 39). Furthermore, constrained healthcare resource allocation, inadequate technical training, and other issues, particularly when technicians lack sufficient training to operate the equipment proficiently, may also contribute to the repeated use of imaging examinations (40). Systemic inefficiencies are exacerbated in resource-constrained settings. In China, provincial variations in pediatric CT doses and redundant scans due to poor inter-institutional image sharing (30% in referral cases) reflect critical optimization targets (41).

The International Atomic Energy Agency (IAEA) has issued a statement urging improved radiation protection for patients undergoing multiple imaging procedures. Advocates calling for strategies to manage clinical situations that warrant frequent imaging procedures have focused particularly on tackling the cases where repeated radiological imaging may increase cumulative radiation doses for patients (42).

### 2.5 Regional variability in healthcare resources and radiation safety practices

The implementation of international guidelines (e.g., IAEA recommendations) and national regulations (e.g., U.S. EPA standards) varies significantly across regions, leading to disparities in radiation protection awareness and practices (43). Additionally, the availability of training and public education programs differs globally, influencing how communities perceive and manage radiation risks (44). These factors contribute to heterogeneous levels of radiation safety efficacy worldwide. In low- and middle-income countries (LMICs), access to advanced radiological facilities is notably limited. For instance, only 70% of Latin American countries and 46% of African nations possess radiotherapy facilities, with resource allocation heavily skewed by income levels (45). LMICs often face challenges in enforcing radiation protection protocols due to insufficient infrastructure and public awareness, thereby incurring increased cumulative exposure risks (46). Conversely, high-income countries with robust healthcare systems tend to implement stricter diagnostic protocols and dose optimization strategies, which ultimately improve radiation safety.

A multinational study highlighted stark discrepancies in cumulative effective doses (CED), revealing that patients in certain regions exceed 100 mSv more frequently than others, underscoring the impact of regional practices (7). Inter-institutional variability further exacerbates this exposure disparity differences in adherence to imaging protocols and quality control measures lead to inconsistent radiation exposure levels among patients undergoing similar procedures (47). For example, hospitals in resource-limited settings may resort to excessive dose reduction due to equipment constraints, while well-resourced institutions adopt advanced dose modulation technologies.

While these disparities pose significant challenges, advancements in imaging technology (e.g., AI-driven dose optimization) and global collaborations (e.g., IAEA’s technical cooperation programs) offer pathways to mitigate inequities. Addressing regional variability requires a multifaceted approach, including policy harmonization, capacity-building initiatives in LMICs, and culturally tailored public education campaigns to foster universal adherence to ALARA principles. Future efforts must prioritize bridging regional disparities in healthcare resources and radiation safety practices through international cooperation, ensuring equitable access to dose optimization technologies and education programs.

## 3 Identification of high-risk populations

Identifying high-risk populations for repeated imaging examinations is essential for optimizing patient care and minimizing unnecessary radiation exposure. Several factors influence the likelihood of repeated imaging, including clinical conditions, patient demographics, and healthcare system practices. These elements interplay to create a nuanced picture of imaging necessity. A comprehensive understanding of these factors facilitates the development of strategies to reduce unnecessary repeated imaging and its associated risks.

### 3.1 Emergency department and ICU patients: cumulative dose in acute situations

Patients in the emergency department (ED) represent a high-risk group for repeated imaging procedures due to the acute nature of their conditions, the complexity of diagnosis, and the urgency of treatment decisions (48–50). Emergency room patients often present with complex conditions that pose diagnostic challenges, and repeat imaging during follow-up visits is frequently associated with diagnostic errors in initial assessments (11). These errors significantly elevate the risk of adverse outcomes, particularly when follow-up imaging is performed within 72 h of the initial examination.

Trauma is a leading cause of repeated imaging in the ED, especially among polytrauma patients (51). These patients typically require multiple imaging studies over a short period due to the severity of their injuries and the complexity involved in assessing the extent of damage. For instance, combined CT scans of the chest, abdomen, and pelvis are frequently employed to evaluate the full extent of injuries in polytrauma patients (50). Studies indicate that patients with higher Injury Severity Scores (ISS) tend to require more imaging examinations, resulting in a higher frequency of repeated assessments (20). Griffey et al. (50) reported that over 50% of ED patients underwent ≥10 CT scans during follow-up, with cumulative effective doses (CED) surpassing 91 mSv, where CT was the predominant source of radiation.

The impact of trauma mechanisms on radiation exposure levels is significant. High-intensity traumas, such as traffic accidents and falls, often result in higher CED, while lower levels of exposure are observed in patients with injuries from slips or animal bites (51). A study by You et al. (51) further revealed that in patients with CED exceeding 100 mSv, the most common causes of trauma were pedestrian injuries, falls, motorcycle accidents, and vehicle collisions. Head trauma is also a major contributor to CT imaging in the ED, especially in patients with severe head injuries (e.g., Glasgow Coma Score < 13), where the diagnostic value of CT outweighs the associated radiation risk. However, routine CT scans for mild head injuries remain controversial (52).

Acute disease patients: this category includes patients presenting with acute conditions such as abdominal pain, chest pain, and cerebrovascular events (52, 53). The rapid need for diagnosis and treatment in these cases often leads to multiple imaging studies, especially when the etiology remains unclear or multiple diagnoses must be considered. For example, patients with acute abdominal pain may undergo CT scans to rule out bowel obstruction or visceral injury. Between 2007 and 2013, approximately 25–30% of emergency department patients with abdominal pain underwent CT scans, according to CDC statistics (54). Among patients with CED ≥ 100 mSv, abdominal pain was identified as the most common clinical indication for CT scans in those without a history of malignancy (53).

Patients with chronic diseases in acute exacerbation: patients with chronic conditions, such as COPD or cardiovascular diseases, may require repeated imaging during acute exacerbations. For example, COPD patients may undergo CT or X-ray to assess for complications such as pneumonia or emphysema during an acute exacerbation. Studies have shown that patients with chronic diseases often experience frequent imaging exams in the ED. Jaffe et al. (55) found that 9% of Crohn’s disease patients at their institution underwent ≥5 abdominal or pelvic CT scans, with 3% of these patients undergoing ≥10 scans, and nearly 50% of these examinations were carried out primarily in the emergency department. This suggests that acute exacerbations of chronic diseases can lead to significant increases in radiation exposure, highlighting the importance of monitoring cumulative radiation doses during long-term follow-up.

In summary, high-risk populations for repeated imaging in the emergency department include trauma patients and those with acute exacerbations of chronic conditions. For these populations, it is important to implement risk stratification assessments in the emergency department (ED) using tools such as the Injury Severity Score (ISS) to identify patients who may need multiple imaging examinations (51). It is essential to balance the urgency of obtaining accurate diagnoses with the need to monitor cumulative radiation exposure. Protocols should be optimized based on cumulative radiation doses to ensure appropriate radiation protection.

Intensive care unit (ICU) patients: ICU patients who require continuous monitoring and support for multiple injuries, organ failure, or complex conditions are at increased risk of cumulative radiation exposure from repeated imaging due to the critical need for diagnostic information and treatment decisions (10). The increasing use of advanced imaging modalities, radiological diagnostics, and interventional radiology in critical care settings has contributed to this trend (8). The frequent reliance on imaging protocols is closely linked to fluctuations in the patient’s condition, diagnostic complexity, and the need to confirm medical device placements.

Severe trauma patients: trauma patients in the ICU are typically admitted due to polytrauma, organ injury, or similar reasons. These patients frequently require repeated imaging, primarily in response to clinical status alterations (56). Whole-body computed tomography (WBCT) is commonly performed during the initial assessment to evaluate the severity of injuries, with follow-up imaging required if the patient’s condition evolves or complications develop (57). Research has shown that trauma patients with higher Injury Severity Scores (ISS) generally require more frequent imaging due to the need for diagnosis and monitoring (26). As a result, imaging frequency is typically greater in trauma patients, particularly those with severe injuries (58).

Critically Ill Patients: Critically ill patients, such as those with severe respiratory failure, acute myocardial infarction, or acute renal failure, typically require frequent imaging examinations due to changes in their condition, the occurrence of complications, and the need to assess treatment efficacy. Disease severity is one of the critical factors influencing the frequency of imaging exams and cumulative effective dose (CED) (26). A study based on the APACHE III score indicates that ICU patients with higher APACHE scores undergo significantly more CT scans, and these scans account for over 90% of the cumulative effective dose (CED) (59).

Monitoring line placement and excluding pneumothorax: critical care practice necessitates frequent placement of central venous catheters, nasogastric tubes, endotracheal tubes, chest drainage tubes, and other lines (8, 60). Furthermore, mechanical ventilation is a foundational therapeutic intervention in critical care, particularly for patients with respiratory failure. Such patients require repeated chest X-rays or CT scans to monitor lung conditions, assess ventilation effectiveness, and rule out complications such as infections or pneumothorax (61).

Length of stay (LOS): length of stay (LOS) is an important factor influencing the frequency of repeated imaging examinations in ICU patients. Research has shown that patients with prolonged hospital stays typically require more imaging, particularly when their condition becomes more complex or deteriorates (62). A prospective study by Hui et al. (63) found that among surgical ICU patients with a LOS exceeding 30 days, the proportion of patients with a cumulative effective dose (CED) >50 mSv significantly increased. Furthermore, the CED in medical ICU (MICU) patients was significantly higher than that of surgical or trauma patients over the short term. Thus, when conducting repeated imaging in the ICU, clinical decision-making should consider the patient’s clinical needs, disease severity, and LOS to ensure that imaging is appropriately planned to avoid unnecessary use and minimize radiation exposure risks (63).

### 3.2 Chronic disease patients: the invisible costs of long-term follow-up

Patients with cardiovascular diseases, especially those who have experienced acute myocardial infarction, undergone heart transplantation, or received endovascular aortic repair (EVAR), often require serial imaging examinations throughout both acute and long-term follow-up phases (64). These imaging modalities serve essential roles in diagnosing cardiovascular abnormalities, monitoring postoperative complications, and evaluating therapeutic outcomes (65). Heart transplant recipients typically require long-term follow-up and imaging surveillance to assess transplanted organ function, exclude rejection, and monitor for complications (65). Post-EVAR patients require regular follow-up to monitor for potential complications, including endogenous, graft displacement, and aneurysm expansion (66).

Individuals with congenital heart disease typically require prolonged and repeated imaging examinations (67, 68). Pediatric populations exhibit increased radiosensitivity, necessitating special consideration. For instance, the HARMONIC project, a multicenter cohort study funded by European institutions, evaluated radiation doses and health impacts of imaging examinations on children and adolescents with congenital heart disease (67). Epidemiological evidence demonstrates that over 10% of newborns and children aged 4–30 months received more than 10 conventional radiographic exams in the past few years, while the frequency of such procedures is comparatively lower in older adult patients.

Quantitative analyses indicate that cumulative effective dose (CED) levels in cardiovascular disease patients are significantly higher than those in the general population. McDonnell et al. (65) found that 91% of the CED comes from catheterization procedures, 31% occurs during the transplant hospitalization, and 62% arises during long-term follow-up. For patients with acute myocardial infarction, Eisenberg et al. (69) found that 18% of patients had a cumulative CED > 30 mSv within the first year after onset.

Patients with pulmonary diseases, such as those undergoing lung cancer screening, diagnosed with pulmonary thromboembolism (PTE), or those who have received lung transplants, often require repeated imaging examinations for diagnosis and follow-up (70). These examinations play a crucial role in disease diagnosis, treatment evaluation, and long-term follow-up. For example, low-dose CT (LDCT) screening, as the standard method for lung cancer screening, has been shown to reduce lung cancer-related mortality significantly (71). PTE patients are primarily diagnosed and assessed through CT pulmonary angiography (CTPA). In contrast, lung transplant recipients require long-term imaging monitoring to assess graft function and rejection (13). These imaging procedures are significant in providing diagnostic information and guiding clinical management; however, prolonged radiation exposure may confer substantial iatrogenic risks, particularly with long-term monitoring or frequent examinations. Research indicates that the cumulative effective dose (CED) levels in these patients are significantly increased compared to the general population, and the associated stochastic effects necessitate comprehensive risk stratification (13).

Patients with renal diseases, including those with urinary stones, end-stage renal disease (ESRD), and kidney transplant recipients, often require repeated imaging examinations for diagnosis, treatment evaluation, and postoperative monitoring. For instance, patients with urinary stones, who have a high recurrence rate of 35–40% within 10 years, often require multiple CT scans for diagnostic confirmation (12). Research indicates that CT, recognized as the “gold standard” for urinary system imaging, demonstrates a sensitivity and specificity exceeding 95% in detecting urinary tract stones (72). However, the high frequency of CT scans significantly increases the cumulative effective dose (CED) in patients with urolithiasis. According to Katz et al. (12), 4% of patients with urinary stones accumulated a CED between 20 and 154 mSv over 6 years, with some individuals reaching levels of radiation exposure warranting close monitoring. For end-stage renal disease (ESRD) patients, their CED levels are even higher. These patients often require repeated imaging examinations due to the frequent occurrence of comorbidities, dialysis-related surgeries, and the need for post-kidney transplant complication monitoring (22). Brambilla et al. (22) estimated that during a three-year follow-up, 16% of hemodialysis (HD) patients accumulated a CED ≥ 100 mSv, with approximately one-third of patients accumulating a CED of 50–100 mSv within 3–4 years. The annual average CED for ESRD patients is over seven times that of background radiation. In contrast, the CED for kidney transplant recipients is slightly lower (five times the background radiation). Still, due to their younger average age (higher proportion of younger patients), the long-term risk of radiation-induced malignancies is higher. Notably, CT scans are the major contributor to CED in ESRD patients, accounting for approximately 66–75% of the total CED. Furthermore, kidney transplant recipients require regular imaging examinations for long-term follow-up to monitor graft function and complications (such as graft rejection and vascular complications). These patients, given their predominance in younger demographic strata, may be at greater risk of radiation-induced health issues in the future and require the implementation of individualized radiation dose optimization protocols.

Patients with inflammatory bowel disease (IBD), especially those with Crohn’s disease (CD) and ulcerative colitis (UC), frequently need multiple imaging examinations for diagnosis and ongoing evaluation due to the chronic disease course and complications such as small bowel obstruction and abdominal pain. CT imaging, as the gold standard for diagnosing small bowel obstruction (SBO), is commonly applied to these patients (7). Studies show that 10–30% of IBD patients accumulate a CED exceeding 50 mSv in imaging examinations, with Crohn’s disease patients having particularly high CED levels (53). Due to the widespread distribution of lesions and the complexity of the condition, Crohn’s disease patients have a mean annual CED exceeding 200% of natural background radiation levels, while UC patients have a relatively lower CED, usually below background radiation levels. This difference is primarily due to the varying severity of the diseases and the differing demands for imaging examinations. It is noteworthy that the average age of IBD patients is relatively low (reported average age between 32 and 46 years), which is clinically significant in terms of radiation-induced health risks for this younger population. Especially for Crohn’s disease patients, who are treated with immunosuppressive agents, the risk of lymphoma and other malignancies is increased, and radiation exposure may further compound these health risks.

### 3.3 Identification of special populations

#### 3.3.1 Identification of pediatric populations at high risk

Adolescent idiopathic scoliosis (AIS) is a progressive spinal deformity that typically emerges during puberty (73, 74). Frequent radiological evaluations during treatment expose scoliosis patients, particularly those undergoing surgery, to higher radiation levels, with cumulative doses often approximately 10 times greater than those associated with conservative treatments (75). In scoliosis management, adolescents, particularly female patients, often require routine spinal X-rays to monitor disease progression, evaluate treatment effectiveness, and conduct follow-up care (76). Prolonged radiation exposure not only increases radiation-related risks but may also contribute to the development of late-stage diseases, including breast cancer. Data indicate that during treatment, scoliosis patients undergo an average of 24.7 imaging examinations, with the associated risk of breast cancer nearly doubling (77). Special attention should be given to the frequency of imaging examinations and the associated radiation burden, particularly for adolescent females, to minimize potential health risks.

Pediatric cancer patients, especially those with solid tumors, frequently require multiple imaging examinations during treatment and follow-up, which are typically associated with low-dose ionizing radiation exposure (74). Although imaging monitoring is believed to enhance overall survival (OS) following pediatric malignancy treatment (78), pediatric cancer patients continue to require multiple imaging evaluations for diagnosis, clinical staging, treatment response assessment, and follow-up monitoring. The cumulative radiation dose from these examinations is significant, and pediatric cancer patients, compared to the general population, exhibit greater sensitivity to low-dose radiation, particularly in relation to harmful effects from CT examinations (78). Special attention should be given to patients undergoing repeated imaging, as frequent scans offering minimal clinical benefit may result in a cumulative risk–benefit imbalance, which justifies the decision to discontinue imaging for the same clinical indications (79).

#### 3.3.2 Identification of female populations at high risk

In chest CT examinations, female breast tissue is invariably exposed to X-ray radiation, potentially increasing the risk of damage to breast tissue (80). Both normal breast tissue and breast cancer tissue exhibit high sensitivity to ionizing radiation, and low dose ionizing radiation in the chest region may elevate the risk of breast cancer in high-risk women. Breast cell proliferation during puberty, pregnancy, and adolescence increases DNA synthesis, rendering breast tissue particularly susceptible to the carcinogenic effects of radiation (81). Female patients frequently require repeated imaging examinations during disease management, particularly in breast cancer screening and follow-up. As the incidence of breast cancer continues to rise, the global demand for long-term monitoring programs has concurrently increased (82). It is important to note that for female patients undergoing frequent imaging examinations, the relative risk of breast cancer increases significantly with high-dose radiation exposure (62). Women with a family history of breast cancer or genetic predisposition are more likely to undergo frequent imaging to detect the disease at an early stage. Studies indicate that women with a family history of breast cancer are more sensitive to ionizing radiation, resulting in a significantly higher frequency of imaging examinations compared to the general population (83).

## 4 From acute to chronic risks: temporal patterns in recurrent medical imaging

The time interval between imaging examinations is critical in determining cumulative radiation effects (19). Current evidence indicates that high-frequency imaging procedures performed within shortened temporal intervals, particularly those involving increased radiation doses, can lead to the rapid accumulation of cumulative radiation exposure. Such recurrent imaging protocols amplify the radiation burden on radiosensitive organs (e.g., hematopoietic system, thyroid gland, and pulmonary parenchyma), significantly increasing the likelihood of acute radiation-induced sequelae (52). Furthermore, short-interval recurrent radiation exposure is significantly correlated with the pathogenesis of specific malignancies, including breast cancer and hematologic neoplasms. As the frequency of imaging examinations increases, cancer risk escalates substantially, particularly with cumulative CT exposures (84). In contrast to acute exposure scenarios, protracted imaging protocols administered with extended intraprocedural intervals are primarily associated with the development of chronic radiation-induced pathologies. For instance, prolonged low-dose radiation exposure, while not inducing acute biological responses, may elevate the risk of chronic conditions, including cardiovascular disorders, cataract formation, and neurocognitive impairments (66). Despite the modest radiation dose per imaging session, the cumulative dose accrued over time may confer an increased risk of carcinogenesis. Research suggests that prolonged and sustained radiation exposure may contribute to the progressive worsening of chronic health conditions (30).

Moreover, although numerous studies have identified the long-term health risks of ionizing radiation from medical imaging, the findings are not entirely consistent. Contemporary biological studies have shown that low-dose radiation may induce beneficial biological responses, including immunostimulant and the upregulation of antioxidative mechanisms (85). The experimental study conducted by Lemon et al. (86) demonstrated that repeated CT imaging post-tumorigenesis may attenuate the progression of specific radiation-induced malignancies in TRP53 + murine models, potentially ameliorating cancer incidence. This finding suggests that while the cumulative effects of radiation exposure are generally significant, in some cases, supra-threshold radiation exposure may compromise tissue protective mechanisms. According to the linear no-threshold (LNT) model, the risk of each radiation exposure is independent, and the single exposure dose is proportional to the risk (87). Based on this assumption, the risks of recurrent medical imaging may not simply accumulate. Contemporary radiobiological research confirms the existence of short-term DNA repair mechanisms, especially in response to double-strand DNA breaks caused by CT imaging. Research by Löbrich et al. (88) shows that double-strand DNA breaks caused by CT imaging can be fully repaired within 24 h, and the post-repair damage level is lower than pre-irradiation levels, providing strong evidence for radiation damage repair. This further suggests that the risks associated with recurrent diagnostic imaging may not follow a linear cumulative pattern, and endogenous repair mechanisms may attenuate short-term health impacts.

Given the distinct characteristics of various diseases and patient groups, the interval between imaging examinations should be assessed according to the individual’s clinical condition and prior examination history. Individuals with congenital heart disease generally necessitate prolonged and multiple imaging assessments, making them a representative group for investigating the long-term health effects of radiation. The multinational HARMONIC consortium study (67), a European-funded multicenter cohort investigation, evaluated the radiation dose and subsequent health effects of imaging procedures in children and adolescents with congenital heart disease. Research by Afroz et al. (67) demonstrated that, while the dose from a single routine radiographic exam is low, frequent examinations markedly augment the overall contribution to the cumulative effective dose (CED). Specifically, chest X-rays and chest CT examinations account for most of the total effective dose.

Furthermore, regarding optimizing chest CT examination practices, Kang et al. (89) analyzed the changes in imaging parameters and protocols before and after the 2008 release of the Korean Chest CT guidelines. They found that dose-reduction strategies, including tube current modulation, demonstrated dual benefits: decreased per-examination radiation burden and enhanced image fidelity. This indicates that optimizing examination techniques and frequencies can significantly reduce the accumulation of unnecessary radiation.

Current studies primarily concentrate on specific imaging modalities or patient populations, with limited research exploring the direct relationship between examination intervals and health outcomes. Most of the existing literature is concentrated on patient groups or individual examination types, with a significant gap in systematic studies addressing diverse populations and varying examination intervals. For example, research on imaging examination intervals tends to rely more on short-term data, with long-term follow-up studies being relatively scarce. Overall, existing studies support the rational optimization of examination intervals to reduce unnecessary radiation exposure and decrease health risks. Especially based on long-term follow-up, more attention must be paid to the reasonable design of examination intervals and personalized radiation protection strategies to maximize diagnostic benefits and reduce health risks.

## 5 Discussion

Radiation risk assessment in medical imaging represents a complex interplay between dosimetrist parameters, biological susceptibility, and epidemiological evidence. Research on the carcinogenic effects of low-dose radiation remains a focal point of international scientific inquiry, particularly in the field of medical imaging. Although the differentiation between radiation-attributable malignancies and spontaneous neoplasms presents significant diagnostic and etiological challenges, existing risk assessment models, such as the Excess Relative Risk (ERR) and Excess Absolute Risk (EAR) models proposed by BEIR VII, have provided fundamental frameworks for quantifying the risk of radiation-induced carcinogenesis, further refining risk assessments (84). According to a 2019 report by the International Atomic Energy Agency (IAEA), an estimated 1 million patients worldwide annually are exposed to cumulative effective doses exceeding 100 mSv due to repeated medical imaging procedures (7). This finding highlights that radiation exposure from recurrent imaging procedures (such as repeated CT scans) may significantly impact cancer risk. Particularly in cases of high cumulative radiation doses, the attributable radiation risk exceeds statistical significance thresholds.

Radiation risk assessment constitutes a systematic process for evaluating the probability and potential consequences of health impacts from radiation exposure, involving the quantification of health risks from radiation exposure. Although diagnostic radiation exposure typically confers minimal individual risk, the substantial population exposed annually means that even small risks can accumulate, leading to widespread health impacts, particularly cancers, from long-term exposure. Risk prediction models integrate large amounts of epidemiological data to assess the long-term cancer risk following radiation exposure (52). Radiation epidemiology plays a crucial role in studying the health impacts of radiation exposure on populations, providing empirical data that informs risk models (90).

Based on the clarified radiation risk assessment models and methods, the next key step is how to apply these theoretical results in clinical practice and optimize technical approaches to effectively reduce the radiation risks that patients face. Although radiation risk assessments provide us with important insights into the relationship between different radiation exposure levels and health effects, these theoretical findings can only be fully realized in actual clinical applications. The translation of theoretical risk assessment into clinical practice necessitates robust technological implementation strategies. Technological optimization is a means to reduce radiation doses and the core of achieving a balance between medical imaging diagnostic accuracy and patient safety. With the rapid advancement of medical imaging technology, how to meet the growing demand for imaging while minimizing unnecessary radiation exposure has become a pressing challenge in global radiological health (91). In response to this challenge, organizations such as the International Commission on Radiological Protection (ICRP) have introduced multiple optimization measures for radiation exposure and have advanced research and the application of related technologies (14, 92).

Recent advancements in imaging technologies, including low-dose protocols, hybrid imaging modalities (e.g., PET/CT), and enhanced digital radiography systems, have significantly reduced radiation doses while maintaining or improving diagnostic accuracy (93). Concurrently, the rapid development of artificial intelligence (AI) and machine learning is revolutionizing medical image analysis, enabling intelligent diagnostics through automated lesion detection, dose optimization, and risk stratification (94). For instance, AI-driven algorithms can tailor imaging protocols to individual patient profiles—such as age, prior exposure history, and clinical indications—thereby minimizing unnecessary radiation in recurrent examinations (95).

The integration of AI into radiation protection strategies facilitates precise dose tracking and predictive risk modeling, which are critical for managing cumulative exposure in high-risk populations (96). Deep learning models outperform traditional manual evaluations in identifying subtle pathological changes, reducing diagnostic errors, and supporting evidence-based clinical decisions. This technological convergence transforms multiple facets of medical practice, from early disease detection and accurate diagnosis to personalized treatment planning and prognostication (97). In emergency care, real-time AI-based analysis of trauma scans prioritizes critical findings, curbing redundant imaging (94).

These innovations underscore the potential to harmonize diagnostic efficacy with radiation safety. However, widespread adoption requires addressing challenges such as interoperability of AI tools, validation across diverse populations, and equitable access to advanced technologies—particularly in resource-limited settings.

## 6 Conclusion

A balanced approach to medical imaging is essential, particularly in the context of recurrent examinations, to mitigate the risks associated with cumulative radiation exposure. Our findings demonstrate that while advanced imaging technologies, such as CT have significantly enhanced diagnostic capabilities, their overuse, particularly in high-risk populations, poses substantial health risks, including increased cancer incidence and other radiation-induced pathologies. We have identified key drivers—from clinical necessity and demographic factors to technological advancements and systemic inefficiencies—providing a comprehensive framework for addressing these challenges.

To minimize unnecessary radiation exposure, it is essential to implement individualized risk stratification and optimize imaging protocols based on patient-specific factors, including age, clinical condition, and prior exposure history. Technological innovations, such as dose modulation and advanced image reconstruction algorithms, provide effective strategies for reducing per-examination doses without compromising diagnostic accuracy. Furthermore, developing robust radiation safety education programs and standardized guidelines for high-risk populations, including emergency department patients, chronic disease cohorts, and pediatric groups, is crucial to ensure adherence to the ALARA principle.

This research also calls for a paradigm shift in clinical practice, as we emphasize the importance of long-term follow-up studies to better understand the temporal patterns of radiation exposure and their associated health impacts. Future studies can refine risk assessment frameworks and inform evidence-based policies by integrating epidemiological data, dosimetrist models, and clinical insights. Ultimately, the findings advocate for a collaborative effort among healthcare providers, policymakers, and researchers to harmonize technological advancements with patient safety, ensuring that the benefits of medical imaging are maximized while minimizing its potential harms.
